# Supplementary material for: Solar system expansion and strong equivalence principle as seen by the NASA MESSENGER mission
Source: Nat Commun. 2018 Jan 18;9:289. doi: 10.1038/s41467-017-02558-1 (PMC5773540; doi:10.1038/s41467-017-02558-1)
Supplement: Supplementary file 1 — Supplementary Information [file 41467_2017_2558_MOESM1_ESM.pdf]

	No Constraints					Nordtvedt Equation				
	$GM_\odot$	$J_{2\odot}$	*β-1*	η	$G\dot{M}_\odot/GM_\odot$	$GM_\odot$	$J_{2\odot}$	*β-1*	η	$G\dot{M}_\odot/GM_\odot$
$GM_\odot$	1	-	-	-	-	1	-	-	-	-
$J_{2\odot}$	0.70	1	-	-	-	0.3	1	-	-	-
β-1	0.70	1	1	-	-	0.35	0.90	1	-	-
η	0.14	-0.02	-0.06	1	-	0.35	0.90	1	1	-
$G\dot{M}_\odot/GM_\odot$	-0.01	-0.02	-0.004	-0.14	1	-0.04	-0.20	-0.14	-0.14	1

Supplementary Table 1 Correlations between heliophysics and relativistic parameters. The correlations of the estimated $GM_\odot$, $J_{2\odot}$, β, η, and $G\dot{M}_\odot/GM_\odot$ were retrieved with (right) and without (left) assuming a priori constraints. The correlation between $J_{2\odot}$ and β decreases to 0.9 if the Nordtvedt equation is applied.

	No Constraints	***Fixed β = 1***	***Fixed η = 0***	***Fixed β = 1 and η = 0***
$GM_\odot$ (km^3 s^{-2})	132712440041.7776±0.52	132712440042.3371±0.47	132712440041.7296±0.51	132712440042.4502±0.3[illegible]
$J_{2\odot}$ (×10^{-7})	2.1052±0.15	2.2657±0.0057	2.1052±0.15	2.2709±0.0044
β-1 (×10^{-5})	-1.43±1.47	0.00	-1.39±1.47	0.00
η (×10^{-5})	-5.48±7.3	-5.89±0.73	0.00	0.00
$G\dot{M}_\odot/GM_\odot$ (×10^{-14} yr^{-1})	-6.147±1.47	-6.152±1.47	-6.060±1.47	-6.31±1.47

Supplementary Table 2 Relativity and heliophysics estimates of four different cases. The first column shows the solution that does not assume a metric theory of gravitation, so no constraint (i.e. Nordtvedt equation) was applied. The other three cases are based on the assumption that β-1, η or both parameters are fixed to 0. These multiple scenarios highlight the stability of $G\dot{M}_\odot/GM_\odot$ and η solutions, which do not rely significantly on the *a priori* assumptions.

	DE432	*DE430*	*DE436*	*Formal Uncertaintie*
$GM_{\odot}$ ($km^3\ s^{-2}$)	132712440042.26	132712440043.05<br>*[+0.790]*	132712440043.13<br>*[+0.870]*	0.35
$J_{2\odot}$ ($\times 10^{-7}$)	2.246	2.225<br>*[-0.021]*	2.237<br>*[-0.009]*	0.02
β-1 ($\times 10^{-5}$)	-1.625	-0.219<br>*[+1.406]*	-0.056<br>*[+1.569]*	1.8
η ($\times 10^{-5}$)	-6.646	-0.874<br>*[+5.772]*	-0.225<br>*[+6.241]*	7.2
$G\dot{M}_{\odot}/GM_{\odot}$ ($\times 10^{-14}\ yr^{-1}$)	-6.130	-9.274<br>*[-3.144]*	-7.079<br>*[+0.949]*	1.47

Supplementary Table 3. Relativity and heliophysics estimated values with three different planetary ephemerides. Different JPL DE ephemerides for the Sun, the Moon, asteroids, and planets were assumed to demonstrate the stability of our relativity and heliophysics results. The first column is the solution reported in Table 2 with JPL DE432. The other two ephemerides used in this study are DE430 and DE436. The red text in parenthesis shows the differences of DE432 estimates with respect to the other two test cases. The DE430 were generated for the GRAIL mission, so they were especially dedicated to the Earth-Moon system. The DE432 represents an enhanced version of the DE430 with improved orbits for Jupiter and Pluto. The DE436 ephemerides, on the other hand, have been recently released for the NASA mission Juno with an update of the ephemeris of Jupiter. The maximum discrepancies in Earth position between DE430 and DE432, and DE436 and DE432 are ~30 m and ~80 m, respectively. In spite of these large differences between these ephemerides, $J_{2\odot}$, β, and η solutions are stable within 1-σ. $GM_{\odot}$ and $G\dot{M}_{\odot}/GM_{\odot}$ estimates show larger variations that are between 2- and 3-σs. Mismodelings in the planets trajectories, including the Earth, and adjustments of the SSB location, which differs, for

example, by ~200 m between DE430 and DE432, mainly affect the accuracy on the estimation of the solar gravitational constant value and time-variation.
